# A single-blind randomised controlled trial of the effects of a web-based decision aid on self-testing for cholesterol and diabetes. study protocol

**DOI:** 10.1186/1471-2458-12-6

**Published:** 2012-01-04

**Authors:** Martine HP Ickenroth, Janaica EJ Grispen, Nanne K de Vries, Geert-Jan Dinant, Glyn Elwyn, Gaby Ronda, Trudy van der Weijden

**Affiliations:** 1 CAPHRI, School for Public Health and Primary Care, Department of General Practice, Faculty of Health, Medicine and Life Sciences, Maastricht University, P.O. Box 616, 6200 MD Maastricht, The Netherlands; 2 CAPHRI, School for Public Health and Primary Care, Department of Health Promotion, Faculty of Health, Medicine and Life Sciences, Maastricht University, P.O. Box 616, 6200 MD Maastricht, The Netherlands; 3 Department of Primary Care and Public Health, Cardiff University, Neuadd Meironnydd, Heath Park, Cardiff CF14 4YS, UK

## Abstract

**Background:**

Self-tests, tests on body materials to detect medical conditions, are widely available to the general public. Self-testing does have advantages as well as disadvantages, and the debate on whether self-testing should be encouraged or rather discouraged is still ongoing. One of the concerns is whether consumers have sufficient knowledge to perform the test and interpret the results. An online decision aid (DA) with information on self-testing in general, and test specific information on cholesterol and diabetes self-testing was developed. The DA aims to provide objective information on these self-tests as well as a decision support tool to weigh the pros and cons of self-testing. The aim of this study is to evaluate the effect of the online decision aid on knowledge on self-testing, informed choice, ambivalence and psychosocial determinants.

**Methods/Design:**

A single blind randomised controlled trial in which the online decision aid 'zelftestwijzer' is compared to short, non-interactive information on self-testing in general. The entire trial will be conducted online. Participants will be selected from an existing Internet panel. Consumers who are considering doing a cholesterol or diabetes self-test in the future will be included. Outcome measures will be assessed directly after participants have viewed either the DA or the control condition. Weblog files will be used to record participants' use of the decision aid.

**Discussion:**

Self-testing does have important pros and cons, and it is important that consumers base their decision whether they want to do a self-test or not on knowledge and personal values. This study is the first to evaluate the effect of an online decision aid for self-testing.

**Trial registration:**

Dutch Trial Register: NTR3149

## Background

Self-tests on body materials are widely available to the general public [1,2]. These tests give consumers the opportunity to test themselves on medical conditions without consulting a health professional first. Self-tests are available for conditions such as diabetes, high cholesterol, kidney disorders, Chlamydia or prostate cancer [1,2]. In a recent study among Dutch Internet users, 18% of the respondents had ever performed a self-test and 66% of all respondents considered to use a self-test in the future [3]. Tests can be performed at home ('home tests'), in which the consumer is responsible for all aspects of the test: performing the self-test, interpretation of the test-result and follow-up behaviour. These tests can be bought on the Internet and are available in drugstores or pharmacies. Other ways of self-testing are 'streetcorner' tests, in which a test is performed by trained personnel in public places (for example in a supermarket), or 'direct access' and 'home collect' tests, in which a sample is taken at a laboratory or at home, is analysed in a laboratory and the results are sent to the consumer by mail or email.

Self-testing does have advantages as well as disadvantages, and the debate on whether self-testing should be encouraged or rather discouraged is still ongoing [4-7]. Self-testing can be considered as an easy way for consumers to gain more insight in their health status, it might lead to early detection of disease, and fits in with our current views on patient autonomy. On the other hand, there are concerns about self-testing, for example whether consumers have sufficient knowledge to enable appropriate use of self-tests, such as insight in indications for testing, and whether they consider the possibility of obtaining false positive or false negative results. Consumers who perform a self-test often perform these tests for reassurance, because the test is offered by an organisation at no costs, or out of curiosity [8,9], and respond quite straightforward to the test result: they generally have a high level of confidence in self-tests, visit a doctor in case of an abnormal test result and are reassured by a negative test result [2,9]. Consumers with an intention towards self-testing also perceive these benefits of self-testing, although they experience several barriers as well, such as doubts concerning the reliability of self-tests and not knowing how to interpret the test result (Grispen et al: An intention to self-testing: a qualitative study regarding consumers' considerations and information needs about self-testing, submitted)

To support consumers in deciding whether they want to perform a self-test or not, objective information on self-tests could be provided to stimulate consumers to weigh the pros and cons of self-testing. Their decision should be based on knowledge as well as personal values, in which these personal values are related to intention, for example, a positive attitude should lead to a positive intention; a so-called informed choice. Consumers with an intention towards self-testing indicated they experienced benefits as well as barriers in self-testing. In other words, they experienced feelings of ambivalence towards self-testing [10]. If these feelings are not resolved and people have to make a decision, they experience feelings of discomfort. Therefore, ambivalent attitude holders have the urge to integrate these feelings into one response; they want to 'choose sides' [10]. To support patients in making a choice that is the best choice in their specific situation, for example for patients facing treatment and screening decisions, decision aids have been developed. These decision aids have shown to improve people's knowledge of options and reduce difficulty in decision making [11].

We developed a web-based decision aid on self-testing http://www.zelftestwijzer.nl, which provides information on self-tests in general, and test specific information on self-tests for diabetes and cholesterol. The web-based decision aid was based on clinical practice guidelines, previous research on self-testing [3,9,12,13], the International Patient Decision Aid Standard (IPDAS) [14,15], and the Health Belief Model (HBM) [16]. The decision aid provides information on self-testing, as well as a value clarification tool to weigh the pros and cons of self-testing. The aim of this study is to investigate the effect of the web-based decision aid on knowledge of self-testing, informed choice, ambivalence and psychosocial concepts.

### Theoretical framework

#### Health Belief Model

The Health Belief Model (HBM) [16] was used as the theoretical framework during the development of the decision aid and will be used in its evaluation as well. According to the HBM, an individual's decision to engage in health-related behaviour is based on their evaluation of the severity of and the susceptibility to a particular condition or illness and the belief that a certain action is effective in reducing their susceptibility to or the severity of this condition. Furthermore, individuals are only inclined to engage in health-related behaviour if they perceive more benefits than barriers associated with that behaviour and if certain cues (for example in self-testing: having a neighbour with diabetes, or the offering of free tests) are present that trigger action. Finally, self-efficacy, the individual's confidence in his or her capability to successfully perform a certain action, is an important concept within the HBM.

Besides the concepts of the HBM, additional concepts of the Theory of Planned Behaviour (TPB) [17] were used in the development and evaluation of the decision aid, namely subjective norm, anticipated regret, moral obligation and response efficacy. These concepts have been shown to contribute to the explanation of health-related behaviour [18-22].

#### Ambivalence

In previous research we found that individuals who intend to perform a cholesterol or diabetes self-test perceived strong benefits as well as strong barriers towards using a self-test (Grispen et al: An intention to self-testing: a qualitative study regarding consumers' considerations and information needs about self-testing, submitted). Individuals who hold both strong positive and negative beliefs or feelings towards a certain action or attitude object are considered to be ambivalent [10]. An important distinction needs to be addressed concerning the ambivalence concept, namely the difference between potential ambivalence [23] and felt ambivalence [24]. Potential ambivalence concerns the coexistence of beliefs that are associated with incongruent evaluations related to a certain behaviour or attitude object. Individuals do not necessarily have to be aware of this incongruence and therefore potential ambivalence can be implicit. Felt ambivalence refers to simultaneously having positive and negative emotions towards a certain behaviour or attitude object. For felt ambivalence to occur, the individual needs to be aware of his conflicting feelings between the two sides of the behaviour or attitude object. These conflicting feelings lead to psychological discomfort which is experienced as being unpleasant. Ambivalent attitude holders are motivated to solve this psychological discomfort by integrating their conflicting feelings in one evaluate response. When this is achieved, ambivalence and the related psychological discomfort are solved [10,25].

### Study aim and research questions

The principal aim of the proposed study is to evaluate the effect of the decision aid on knowledge of on self-testing. The following research questions were formulated:

1. Do consumers who have been exposed to the decision aid have more knowledge of self-testing than the control group?

2. Do consumers who have been exposed to the decision aid more often make an informed choice in self-testing than consumers in the control group?

3. Do the intervention and control groups differ in level of attitudinal ambivalence?

4. What is the effect of the decision aid on the psychosocial factors that predict self-test use for diabetes and cholesterol?

5. Is there a difference between the intervention and control group in follow-up behaviour 3 months after the use of the decision aid?

## Methods/Design

### Study design

A single-blind randomised controlled trial in which the online decision aid will be compared to short, non-interactive information on self-testing in general (non-test-specific). The entire trial will be conducted online.

### Ethical approval

The study was reviewed by The Medical Ethical Committee of Maastricht University Medical Centre. They had no objection to the study proceeding and because the study evaluates usual care and no patient recruitment was required, formal approval was not deemed necessary.

### Setting and participants

Participants will be recruited from an existing Internet panel in the Netherlands that is managed by Flycatcher, an ISO-certified institute for online research associated with Maastricht University. Currently, the panel consists of approximately 14,000 active members between 12 and 96 years of age http://www.flycatcher.eu. Members of the panel are recruited online, by written invitation, face to face contacts or by intermediaries. All individuals aged 12 years or older and who have an email address can apply for the panel. Compared with the Dutch population, the panellists are younger, have a higher level of education and are more often female. The total panel is representative of the Dutch Internet population. Panel members receive invitations to participate in online questionnaires approximately eight times per year, and receive an incentive when they have completed a certain number of questionnaires. Variables of the panel members such as age, sex and level of education are provided by Flycatcher.

### Inclusion and exclusion criteria

Panel members aged 18 or older, with an intention to use a diabetes and/or a cholesterol self-test in the future will be invited to participate in the randomised controlled trial. An intention will be defined as a consumer indicating to maybe, probably, or definitely intending to use a self-test in the future. People who report that they are already diagnosed with diabetes and/or a cardiovascular disease will be excluded from this study.

### Randomisation

Participants will be assigned to one of two groups (having an intention towards doing a cholesterol or towards doing a diabetes self-test). If participants have an intention to do both, they will be assigned to the test towards which they have the strongest intention. Within each group, randomisation over experimental conditions (and invitation to view either the decision aid or the control condition) will be performed by Flycatcher using SPSS. Participants will be blinded for randomisation.

### Intervention

The HBM [16], IPDAS criteria [14,15] and the results of previous quantitative and qualitative studies [3,9,12,13] provided input for the development of the decision aid http://www.zelftestwijzer.nl. The medical information in the decision aid is based on current clinical practice guidelines on screening for diabetes and cardiovascular disease. After the first versions of the decision aid had been developed, the content and usability of the decision aid were assessed by professionals as well as by end users. An iterative procedure was used to improve the decision aid. The core components of the decision aid are described in Table 1. Respondents in the intervention group will be asked to view the decision aid.

**Table 1:** Core components of the decision aid

Homepage	The homepage gives an introduction on how to use the website, and an explanation about the contents of the website. Visitors are asked to read the disclaimer, and a warning is given that the information provided on the website is not suitable for people who are already being treated for diabetes or cardiovascular disease.
General information on self-testing	General information on self-testing, including information on the different kinds of self-tests, the reliability of self-tests in general and eleven cues what to check before doing a self-test.

Information on cholesterol self-testing	Information on cholesterol self-testing: information on risk factors for cardiovascular disease, the role of cholesterol as a risk factor in cardiovascular disease, an interactive tool to determine personal risk for cardiovascular disease, information on the different kinds of cholesterol tests (tests for total cholesterol and HDL cholesterol), how these self-tests have to be performed, and what to do with the test result, including advise when to see a doctor, and information on lifestyle changes.

Information on diabetes self-testing	Information on diabetes self-testing, similar to the information related to the cholesterol self-test, including an interactive tool to assess your personal risk for developing diabetes.

FAQ	Frequently asked questions

Value clarification tool	An interactive tool to weigh the pros and cons of self-testing. This tool first shows 12 propositions on self-testing, of which six represent advantages of a self-testing, and the other 6 the disadvantages of self-testing. Examples of these propositions are: 'I think it is an advantage that I can do the test whenever I want to', or 'I think that it is a disadvantage of self-testing that I have to get a blood sample myself'. People can indicate whether they agree, disagree or feel neutral about these propositions. When they indicate they agree with a proposition, the weighing scales depicted next to the propositions starts shifting towards doing a self test or not doing a self-test. After respondents have filled out all the propositions, they are asked which propositions are most important to them. They are explained that the tool is not meant to give an advise on whether to do a self-test or not, but is aimed at clearing out their personal values on self-testing.

	Sitemap, disclaimer and contact information.

### Control condition

A placebo control condition was designed consisting of general information on self-testing. An existing control condition was not available, since there is no usual care in self-testing. Our intervention is the first decision aid that aims at people with an intention towards self-testing, and, to the best of our knowledge, is the first non-commercial website that provides test specific information on cholesterol and diabetes self-testing. The control condition will consist of a pdf file of one page which gives general information on self-testing (definition of a self-test and the types of self-tests that are available). It does not include test specific information or interactive elements that are included in the original decision aid since these are distinguishing features of our decision aid.

### Methods of data collection

**Figure 1:**
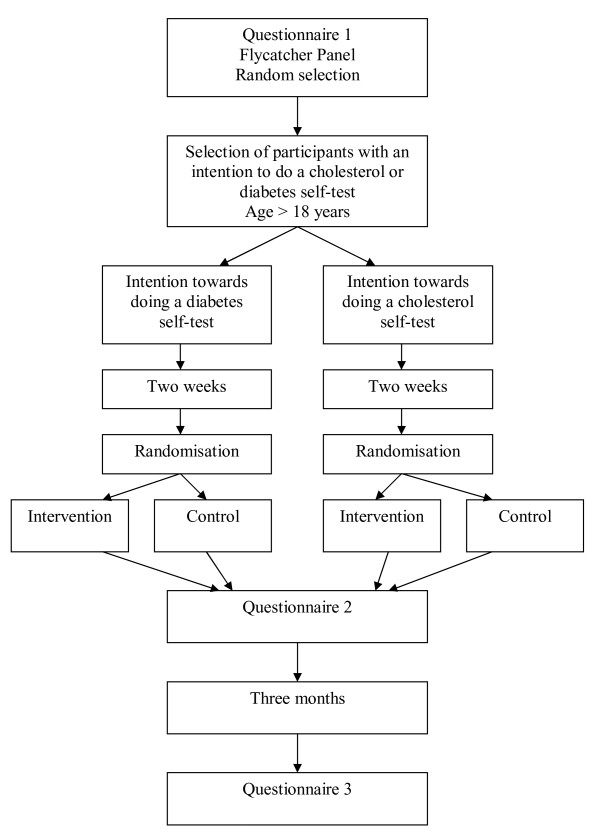
**Selection of participants and randomization**.

An overview of all questionnaires and measures is provided in Table 2 and Figure 1.

**Table 2:** Outcome measures and timing of data collection

Construct	Measures	No. of items	Questionnaire*
*Primary outcomes*			
Knowledge	Knowledge questionnaire based on information provided in the decision aid	20 Statements (True/false/don't know)	2
*Secondary outcomes*			
Attitude	Marteau [26]	4	1,2
Ambivalence	Felt ambivalence [24]	3	1,2
Follow-up behaviour	Uptake of self-tests, visiting a doctor, changing lifestyle.	7	3
Intention	Intention towards self-testing, seeing a doctor.	2	1,2,3
	Intention towards changing lifestyle	1	2,3
	Stages of change [27]	1	1,2,3
Psychosocial determinants	Perceived benefits and barriers	14	2
	Self-efficacy	3	2
	Response-efficacy	4	2
	Perceived susceptibility/risk perception [28]	3	1,2,3
	Cues to action	1	2
	Perceived severity	1	2
	Anticipated regret	1	2
	Moral obligation	2	2
	Subjective norm	1	2

*Questionnaire 1: baseline. Questionnaire 2: directly after seeing intervention or control condition. Questionnaire 3: three months after viewing intervention or control condition

Questionnaire 1 will be sent to a random sample of the Flycatcher panel and is aimed at selecting consumers with an intention towards self-testing for diabetes or cholesterol. The questionnaire will consist of questions on personal characteristics (including medical history of cardiovascular disease and diabetes, and risk factors), and intention towards doing a cholesterol or diabetes self-test. (Additional file 1). A translation of the questionnaires (originally in Dutch) is provided in the additional data files.

Questionnaire 2 will be sent after participants have been assigned to the intervention or control condition and have been exposed to this condition. Participants with an intention to do a cholesterol self-test will receive questions on cholesterol self-testing (Additional file 2), participants with an intention to do a diabetes self-test will receive a similar questionnaire, but addressing the diabetes self-test (Additional file 3).

Three months after participants have viewed the decision aid or the control condition, they will be sent questionnaire 3 (Additional file 4), which includes questions on self-test use in the past 3 months, intention towards doing a self-test, visiting a doctor or changing lifestyle.

### Measures

#### Primary outcome

##### Knowledge

Knowledge questions are based on the themes that are addressed in the decision aid. It includes questions on the condition, indications for testing, such as symptoms and risk factors, what the test measures, how to interpret the test result, and questions on the validity of the self-test. These aspects of self-testing were deemed important by health professionals as well as indicated by end-users [9](Grispen et al: Quality and use of consumer information provided with home test kits: Room for improvement, submitted). This knowledge quiz consists of 20 true/false statements. Knowledge questions are test specific, and differ between the cholesterol and diabetes self-test.

#### Secondary outcome measures

##### Intention

Intention to do a self-test in general and the intention to do a self-test for diabetes and cholesterol. Intention will be assessed with a single question using a 5-point Likert scale with answering options ranging from 1 = 'definitely not' to 5 = 'definitely will'. Further, intention to visit a doctor and intention to change lifestyle will be assessed.

Intention will also be measured using the stages of change algorithm [27]. Stages of change will give more detailed insight in the strength of the participants' intention towards doing a self-test and will be measured by asking participants in what time frame they think they will perform a self-test.

##### Attitude

Attitude towards self-testing for diabetes or cholesterol will be measured using a four item scale developed by Marteau [26]. Participants are asked to respond to the following questions on a 7-point Likert scale: I think self-testing is: harmful--beneficial; unimportant--important; bad thing--good thing; unpleasant--pleasant. Responses are used to classify people as having a positive or a negative attitude. Scores range from 4 to 28, with higher scores referring to a more positive attitude towards self-testing. Whether the participant has a positive or negative attitude towards self-testing will be determined using the median of the scores of the participants [26].

##### Ambivalence

Felt ambivalence will be assessed using a 3 item scale as described by Priester and Petty [24].

##### Psychosocial determinants

Possible determinants of self-test use were derived from the Health Belief Model (HBM) [16], as well as several concepts of the Theory of Planned Behavior (TPB) [17] and will be assessed after participants have viewed the decision aid or control condition.

##### Behaviour

Behaviour will be measured 3 months after the intervention. Participants will be asked whether they have performed a self-test for cholesterol or diabetes, and whether they have visited a doctor or have changed their lifestyle in the past 3 months, in response to having viewed the decision aid. If they have performed a self-test, they will be asked what the result of the self-test was (normal test result, abnormal test result) and the actions taken based on this test result.

### Process evaluation

Message acceptance will be measured on an eight item scale, for example, 'The information provided on the website is very realistic/realistic/neutral/not realistic/not realistic at all' [29]. Participants will be asked to comment on the strong and weak features of the decision aid. Weblog files of each participant will be collected to assess use of the website, time spent on the website and use of each page of the website.

### Sample size

According to a Cochrane review by O'Connor et al. on the efficacy of decision aids, there is an average absolute increase of 15% in knowledge scores when decision aids are compared to usual care. When more detailed decision aids are compared to simpler decision aids, knowledge increases with 5% [11]. Since we will compare our decision aid to general, non test specific information on self-testing, we expect an increase in knowledge of 15% (intervention group knowledge score 70%, control group 55%). Our sample size will be based on a power of 80% to detect a hypothesized increase in knowledge of 15% at a significance level of 5% (two-sided). Power analysis showed 175 respondents would be needed in each of the four groups [30]. Based on previous research, a completion rate of 60% on the first questionnaire, and 80% on the second questionnaire is expected [3]. Intention towards doing a cholesterol and/or a diabetes self-test in 2008 was 42%. Based on these results, a minimum of 3472 respondents would have to be invited for the first questionnaire. Since we do not know intention rate in 2011, and an unknown number of participants will be excluded because of having cardiovascular disease or diabetes, we decided to invite 6000 panel members for the first questionnaire.

### Statistical analysis

Knowledge will be treated as primary outcome measure, whereas intention, attitude, ambivalence and psychosocial variables are the secondary outcome measures. To compare knowledge levels and level of ambivalence between the intervention and control group, we will use linear regression analysis. The effect of the decision aid on the psychosocial factors that predict self-test use will be assessed using logistic regression analysis. Follow-up behaviour after 3 months will be compared using logistic regression analysis.

Based on the method proposed by Marteau and colleagues, informed choice will be measured by combining the constructs knowledge, attitudes and behaviour into a composite measure of informed decision making [26]. Informed choice in our study will be defined as having sufficient knowledge, and attitude being in line with intention (positive attitude and a positive intention to do a self-test, or a negative attitude and a negative intention to self-test). As a cut off point for having sufficient knowledge, a score of 50% (number of correct answered questions 10 or above) will be considered. Individual scores will be corrected for guessing using Abbotts' formula [31,32]. A positive intention towards self-testing is defined as a participant indicating to maybe, probably or definitely consider performing a self-test in the future. To classify participants as having a positive or negative attitude towards self-testing, the median score will be used. The median score of the participants and scores above will indicate a positive attitude, whereas scores below the median score will be considered as a negative attitude [26]. The percentage of people with an informed choice in the control and intervention group will be compared using logistic regression analysis.

## Discussion

This paper describes the protocol for a randomised controlled trial to evaluate the effect of an online decision aid for self-testing on diabetes and cholesterol. Since self-testing on these conditions can be beneficial but can have negative implications as well, the goal of the decision aid 'zelftestwijzer' was not to increase or decrease uptake of these tests, but to enhance knowledge levels and to guide the decision making process. Self-testing uptake in the past often seemed to be associated with offering of free tests and media campaigns stressing the importance of testing, and consumers responding quite straightforward, being curious about testing and wanting to be reassured on their health status [9]. Although these strategies seemed to increase uptake levels, consumers will often not have considered important pitfalls of self-testing [3]. Also, consumers with an intention towards self-testing experience several barriers to self-testing, especially a lack of knowledge on interpreting the results (Grispen et al: An intention to self-testing: a qualitative study regarding consumers' considerations and information needs about self-testing, submitted). For this reason, the primary outcome of this study is knowledge of on self-testing. We expect knowledge levels of participants who have viewed the decision aid to increase. Being aware of the pitfalls of self-testing, but also of the positive effects of self-testing, consumers should be enabled to make a choice regarding self-testing they think is the best choice for them. We expect levels of informed choice to increase, and levels of ambivalence to decrease. We will measure actual uptake of self-tests 3 months after viewing the decision aid, to assess if there are changes in uptake of tests between the intervention and the control group.

### Strengths and limitations

The online decision aid has been developed in a thorough, iterative process, and is considered to be a sound and user-friendly provider of information on self-testing. By using an Internet panel, the online decision aid can be tested among a large group of individuals with an intention towards self-testing. The use of individual weblog files will provide the opportunity to assess how long each participant has viewed the decision aid, and which pages of the decision aid they have visited.

Most decision aid evaluation studies compare the decision aid to usual care, or to simpler versions of the decision aid. Since there is no usual care in self-testing, we decided to provide the control group with a control intervention that consists of only general information on self-testing, without any specific information on diabetes and cholesterol. This minimal information is the same information as was already provided in the instruction section of the baseline questionnaire, so this is the maximum contrast in information provided between intervention and control group possible. The fact that the participants were blind for the trial conditions is a strength, minimising the Hawthorne effect.

The measure of informed choice often includes actual behaviour instead of intention. In our study, measuring behaviour (uptake of tests after seeing the information) would ask for selecting consumers who are at the point of purchasing a self-test. Since self-tests are provided in several websites and shops, it would be almost impossible to gather a group of consumers large enough for a trial. Besides these practical reasons, the fact that we invited consumers who are still considering doing a test, instead of being in a shop, at the at the point of purchasing the test, gives consumers more time to read information on self-testing and thoroughly consider the pros and cons of these tests. Therefore, we chose to invite consumers with a positive intention towards self-testing to view the decision aid, although we know these people are not actually faced with a decision that needs to be made.

Inviting consumers who are not faced with an actual decision that needs to be made can affect the generalisability of our data. Participants in our trial might read other information on the decision aid than consumers who are close to buying a self-test. On the other hand, participants are encouraged to read the complete decision aid, and know they will receive an incentive if they have viewed the decision aid and have filled out questions in the questionnaire provided afterwards. The actual use of the decision aid will also be affected by its usability and attractiveness to visitors. Although the decision aid was developed using usability tests to assess user friendliness of the decision aid, the results of the process evaluation such as information on message acceptance and weblog files, will be of use for optimising and implementing the decision aid after the trial.

## Abbreviations

HBM: Health belief model; IPDAS: International patient decision aids standard; ISO: International organisation for standardisation; TPB: Theory of planned behaviour.

## Competing interests

The authors declare that they have no competing interests.

## Authors' contributions

MI and JG are involved in the study design, the development of the questionnaires and drafted the manuscript. MI and JG contributed equally substantially to the production of this manuscript. NdV, GJD and GE critically revised the manuscript and provided valuable theoretical and design suggestions. GR and TvdW conceived of the study, participated in the study design, and helped to draft the manuscript. All authors have read and approved the final version of the manuscript.

## Pre-publication history

The pre-publication history for this paper can be accessed here:

http://www.biomedcentral.com/1471-2458/12/6/prepub

## Supplementary Material

Additional file 1 **Questionnaire 1 (translated from Dutch)**. Click here for file

Additional file 2 **Questionnaire 2 Cholesterol (translated from Dutch)**. Click here for file

Additional file 3 **Questionnaire 2 Diabetes (translated from Dutch)**. Click here for file

Additional file 4 **Questionnaire 3 (translated from Dutch)**. Click here for file
