# Supplementary material for: A single-blind randomised controlled trial of the effects of a web-based decision aid on self-testing for cholesterol and diabetes. study protocol
Source: BMC Public Health. 2012 Jan 4;12:6. doi: 10.1186/1471-2458-12-6 (PMC3298527; doi:10.1186/1471-2458-12-6)
Supplement: Additional file 1 — Questionnaire 1 (translated from Dutch). [file 1471-2458-12-6-S1.PDF]

Questionnaire 1

The questionnaire was originally in Dutch and has been translated into English

More and more tests are becoming available that allow you to examine your own health. In other words, tests that don't have to be ordered by a doctor. Examples of these tests are tests for diabetes, high cholesterol, kidney disease or Chlamydia. In our survey we try to investigate the use of self-tests en find out what factors may influence their use.

What do we mean by self-tests in this survey?
By self-test we mean a test on body samples (such as blood, urine, faeces or saliva) that can be used to detect a disease or the risk of getting a disease, and which you carry out, or have carried out, at your own initiative (so not on the advice of your own doctor).

What do we NOT consider a self-test in this survey?

- A blood pressure meter is not a self-test in this sense, as it uses no body materials.
- Pregnancy tests are excluded from this survey since pregnancy is not a disease.
- A glucose test which you use at home to monitor your blood sugar levels because you have diabetes is NOT considered a self-test, because this is not aimed at detecting a disease. The disease is already known.
- Asking your doctor to perform a test is NOT considered a self-test because you have a consultation with your doctor before doing the test. We do ask questions about this later on.

There are several ways to use self-tests:

1. buying a self-test for home use from a chemist, pharmacy or supermarket, or ordering it by post, via the Internet or a newspaper or magazine coupon, then applying the test yourself en reading the results;
2. visiting some facility (which may also be a supermarket) at your own initiative, having a test done there and getting the results immediately;
3. visiting a laboratory (e.g. at a hospital) at your own initiative, having a test done there, and getting results sent to you by post;
4. sending in a body sample to a laboratory (at your own initiative), where they do a test and send you the results by post.

It is important that the decision to have the test was taken by you, and not by your doctor.

Questions about self-tests

1. Before this survey, had you ever heard of self-tests? (if yes, multiple answers allowed)

- ☐ yes, I'd heard of self-tests for home use
- ☐ yes, I'd heard about visiting a facility, having a test done there, and getting the results immediately
- ☐ yes, I'd heard about visiting a laboratory to have a body sample taken, and getting the results sent to me by post
- ☐ yes, I'd heard about sending in a body sample to a laboratory, and getting the test results sent to me by post
- ☐ no (respondent is referred to question 4a)

2. Have you ever considered using a self-test? (if yes, multiple answers allowed)

- ☐ yes, a self-test for home use
- ☐ yes, visiting a facility, having a test done there and getting the results immediately
- ☐ yes, visiting a laboratory to have a body sample taken, and getting the results sent to me by post
- ☐ yes, sending in a body sample to a laboratory, and getting the test results sent to me by post
- ☐ no (respondent is referred to question 4a)

3a. Have you ever done a self-test / had a self-test done? (if yes, multiple answers allowed)

- ☐ No
- ☐ Yes, namely…..
  - ☐ Diabetes
  - ☐ Cholesterol
  - ☐ Allergies (hay fever, asthma, house dust mite, food allergies)
  - ☐ Urinary tract infection
  - ☐ Aids / HIV
  - ☐ Anaemia (haemoglobin, hb)
  - ☐ Ovulation
  - ☐ Chlamydia
  - ☐ Glandular fever
  - ☐ Hepatitis B or C
  - ☐ Female fertility or menopause
  - ☐ Male fertility
  - ☐ Syphilis
  - ☐ Vaginal infection (Candida, vaginitis)
  - ☐ Kidney diseases
  - ☐ Thyroid diseases
  - ☐ Influenza
  - ☐ Blood coagulation
  - ☐ Intestinal cancer
  - ☐ Prostate cancer (PSA)
  - ☐ Cervical cancer (HPV)
  - ☐ Helicobacter pylori
  - ☐ Gluten intolerance (celiac disease)
  - ☐ Pharyngitis
  - ☐ Liver diseases
  - ☐ Osteoporosis
  - ☐ Hereditary diseases
- ☐ Other tests, namely…….

3b. When was <u>the last time</u> you did this/these self-test(s)?

	Less than 6 months ago	Between 6 moths and one year ago	Between 1 and 2 years ago	More than 2 years ago
Cholesterol			○	○
Diabetes			○	○
etc. [only the answers to question 3a are shown in the table]			○	○

3c. Where did you buy / have this/these self-test(s)?

	Test for home use	Had it done at supermarket, chemist's, pharmacy or sports centre (e.g. in a screening van)	Visited laboratory to have sample taken	Sent in body sample to a laboratory	Other
cholesterol	○	○	○	○	○
diabetes	○	○	○	○	○
etc. [only answers from question 3a listed in table]	○	○	○	○	○

4a. Do you intend to use self-tests in the future?
- ☐ Definitely not (respondent is referred to question 7)
- ☐ Probably not (respondent is referred to question 7)
- ☐ Perhaps
- ☐ Probably
- ☐ Definitely

4b. What self-test(s) would you consider? (multiple answers allowed)
- ☐ Diabetes (respondent is referred to question 5)
- ☐ Cholesterol (respondent is referred to question 6)
- ☐ Allergies (hay fever, asthma, house dust mite, food allergies) (for all other options, respondents are referred to question 7)
- ☐ Urinary tract infection
- ☐ Aids / HIV
- ☐ Anaemia (haemoglobin, hb)
- ☐ Ovulation
- ☐ Chlamydia
- ☐ Glandular fever
- ☐ Hepatitis B or C
- ☐ Female fertility or menopause
- ☐ Male fertility
- ☐ Syphilis
- ☐ Vaginal infection (Candida, vaginitis)
- ☐ Kidney diseases
- ☐ Thyroid diseases

- Influenza
- Blood coagulation
- Intestinal cancer
- Prostate cancer (PSA)
- Cervical cancer (HPV)
- Helicobacter pylori
- Gluten intolerance (celiac disease)
- Pharyngitis
- Liver diseases
- Osteoporosis
- Hereditary diseases
- Other tests, namely…….

If diabetes is ticked at 4b, respondent is referred to question 5:

You have indicated that you intend to do a self-test for diabetes. The following questions are specifically aimed at this test.

5a. How certain are you that you will do a diabetes self-test?
- Definitely not (respondent is referred to question 7)
- Probably not (respondent is referred to question 7)
- Perhaps
- Probably
- Definitely

5b. When would you [perhaps / probably / definitely; tailored on the basis of question 5a] want to do this self-test?
- Within the next month
- Within the next 6 months (but not within the next month)
- Within the next year (but not within the next 6 months)
- Within the next 5 years (but not within the next year)
- Some time in the future (but not within the next 5 years)
- None of the above

5c. What type(s) of self-test would you consider? (multiple answers allowed)
- A self-test for home use
- Visiting a facility, having a test done there, and getting the results immediately
- Visiting a laboratory to have a body sample taken, and getting the results sent to me by post
- Sending in a body sample to a laboratory, and getting the results sent to me by post

5d. According to you, what are the chances that you will develop diabetes?
- Very high
- High
- Not high / not low
- Low
- Very low
- I already have diabetes

If 'I already have' is ticked at 5d, respondent is referred to question 7

5e. according to you, what are the chances that you will develop diabetes compared to others of your age and gender?

- Much larger
- Larger
- Equally large / small
- Smaller
- Much smaller

5f. To what extent do you feel worried about developing diabetes in the future?

- Not worried at all
- Not worried
- Neutral
- Worried
- Very worried

We now present a number of statements about self-tests for diabetes. What we want to know is you personal opinion, what you personally think about it. Please click the option that corresponds most closely to your opinion.

5g. To me, a diabetes self-test is….

Harmful	○	○	○	○	○	○	○	Beneficial
Unimportant	○	○	○	○	○	○	○	Important
Bad thing	○	○	○	○	○	○	○	Good thing
Unpleasant	○	○	○	○	○	○	○	Pleasant

5h. The following questions are about the way you feel about a diabetes self-test

**As regards doing a diabetes self-test I have …….**								
Very definite feelings	○	○	○	○	○	○	○	Very mixed feelings
**As regards doing a diabetes self-test I experience ….**								
No conflicting feelings at all	○	○	○	○	○	○	○	Highly conflicting feelings
**As regards doing a diabetes self-test I feel…..**								
No doubts at all	○	○	○	○	○	○	○	Very serious doubts

If cholesterol is ticked at 4b, respondent is referred to question 6:

You have indicated that you intend to do a self-test for cholesterol. The following questions are specifically aimed at this test.

6a. How certain are you that you will do a cholesterol self-test?

- Definitely not (respondent is referred to question 7)
- Probably not (respondent is referred to question 7)
- Perhaps
- Probably
- Definitely

6b. When would you expect to [perhaps / probably / certainly; tailored on the basis of question 5a] do this self-test?

- Within the next month
- Within the next 6 months (but not within the next month)
- Within the next year (but not within the next 6 months)
- Within the next 5 years (but not within the next year)
- Some time in the future (but not within the next 5 years)
- None of the above

6c. What type(s) of self-test would you consider? (multiple answers allowed)

- A self-test for home use
- Visiting a facility, having a test done there, and getting the results immediately
- Visiting a laboratory to have a body sample taken, and getting the results sent to me by post
- Sending in a body sample to a laboratory, and getting the results sent to me by post

6d. According to you, what are the chances that you will develop a cardiovascular disease? (cardiovascular diseases are for instance: heart attack, cerebral infarction, CVA, stroke, vascular constriction, claudication, percutaneous angioplasty)

- Very high
- High
- Not high / not low
- Low
- Very low
- I already have a cardiovascular disease

If 'I already have' is ticked at 5d, respondent is referred to question 7

6e. According to you, what are the chances that you will develop a cardiovascular disease compared to others of your age and gender?

- Much larger
- Larger
- Equally large / small
- Smaller
- Much smaller

6f. To what extent do you feel worried about developing cardiovascular disease in the future?

- Not worried at all
- Not worried
- Neutral
- Worried
- Very worried

We now present a number of statements about cholesterol self-tests. What we want to know is you personal opinion, what you personally think about it. Please click the option that corresponds most closely to your opinion.

6g. To me, a cholesterol self-test is….

Harmful	○	○	○	○	○	○	○	Beneficial
Unimportant	○	○	○	○	○	○	○	Important

Bad thing	○	○	○	○	○	○	○	Good thing
Unpleasant	○	○	○	○	○	○	○	Pleasant

6h. The following questions concern the way you feel about a cholesterol self-test

**As regards doing a cholesterol self-test, I have…….**								
Very definite feelings	○	○	○	○	○	○	○	Very mixed feelings
**As regards doing a cholesterol self-test I experience ….**								
No confliciting feelings at all	○	○	○	○	○	○	○	Highly confliciting feelings
**As regards doing a cholesterol self-test I feel…..**								
No doubts at all	○	○	○	○	○	○	○	Very serious doubts

7. Do you intend to ask your family doctor to do a test (or do the same test again) in the future?

- ☐ Certainly not
- ☐ Probably not
- ☐ Possibly
- ☐ Probably
- ☐ Certainly

7b. What test(s) would you consider? (multiple answers allowed)

- ☐ Diabetes
- ☐ Cholesterol
- ☐ Allergies (hay fever, asthma, house dust mite, food allergies)
- ☐ Urinary tract infection
- ☐ Aids / HIV
- ☐ Anaemia (haemoglobin, hb)
- ☐ Ovulation
- ☐ Chlamydia
- ☐ Glandular fever
- ☐ Hepatitis B or C
- ☐ Female fertility or menopause
- ☐ Male fertility
- ☐ Syphilis
- ☐ Vaginal infection (Candida, vaginitis)
- ☐ Kidney diseases
- ☐ Thyroid diseases
- ☐ Influenza
- ☐ Blood coagulation
- ☐ Intestinal cancer
- ☐ Prostate cancer (PSA)
- ☐ Cervical cancer (HPV)
- ☐ Helicobacter pylori
- ☐ Gluten intolerance (celiac disease)

- Pharyngitis
- Liver diseases
- Osteoporosis
- Hereditary diseases
- Other tests, namely…….

Here are some more questions about your health status, lifestyle and personal details.

Health status

8. How would you rate your own health?
- Very poor
- Poor
- Reasonable
- Good
- Very good

9. Do you have one or more of the following conditions?
- Yes, cardiovascular disease
- Yes, diabetes
- Yes, high blood pressure
- Yes, high cholesterol
- None of the above
- I prefer not to answer this question

10. Do you smoke?
- No, I did never smoke
- Yes, I smoked, but I do not smoke anymore
- yes

11. Did your father, mother, sister or brother have a cardiovascular disease before they were 65 years old? *(cardiovascular diseases are for instance heart attack, cerebral infarction, CVA, stroke, vascular constriction, claudication, percutaneous angioplasty)*
- Yes
- No
- I prefer not to answer this question

12. Does your father, mother, sister or brother suffer from diabetes?
- No
- Yes, my father and/or mother
- Yes, my sister(s) and/or brother(s)
- Yes, my father and/or mother as well as my sister(s) and/or brother(s)
- I prefer not to answer this question

13. Are you using medication to control high blood pressure?
- Yes
- No
- I prefer not to answer this question

Question 14 only for women

14. Did you ever have pregnancy diabetes (gestational diabetes)?
- Yes

- o No
- o Not applicable
- o I prefer not to answer this question

15. What is your height (in centimetres)?
… cm [respondents can enter a maximum of 3 digits between 100 and 250]

16. What is your weight (in kilogrammes)?
… kg [respondents can enter a maximum of 3 digits between 0 and 999]

17. What nationality do you have?
- ☐ Dutch
- ☐ Belgian
- ☐ German
- ☐ Moroccan
- ☐ Surinam
- ☐ Turkish
- ☐ Other: …
