# Supplementary material for: A single-blind randomised controlled trial of the effects of a web-based decision aid on self-testing for cholesterol and diabetes. study protocol
Source: BMC Public Health. 2012 Jan 4;12:6. doi: 10.1186/1471-2458-12-6 (PMC3298527; doi:10.1186/1471-2458-12-6)
Supplement: Additional file 2 — Questionnaire 2 Cholesterol (translated from Dutch). [file 1471-2458-12-6-S2.DOC]

**Second questionnaire Cholesterol**

**The questionnaire was originally in Dutch and has been translated into English**

#### **Text and question 1 only for intervention group**

**Some time ago you filled in a questionnaire in which you indicated that you would [Dynamic representation: perhaps, probably, definitely] want to do a cholesterol self-test in the future. That is what this questionnaire is about. Before you start filling in the questionnaire, we want to invite you to visit a website.**

**The link shown below will open the site in a new window. After having visited the site, you can close the window and can start filling in the questionnaire by clicking the “Next” button below.**

**Please click the link shown below to go to the website.**

**Link**

**You have just been shown some information about self-tests. This questionnaire presents you with some questions about the information you have just seen. It also asks you some questions about cholesterol self-tests and certain aspects that may relate to it.**

**1a. Do you think the information on the website** was….

|  | | | | |
| --- | --- | --- | --- | --- |
| Not at all convincing  ○ | Not convincing  ○ | Neutral  ○ | convincing  ○ | Very convincing  ○ |
| Not at all relevant  ○ | Not relevant  ○ | Neutral  ○ | Relevant  ○ | Very relevant  ○ |
| Not at all realistic  ○ | Not realistic  ○ | Neutral  ○ | Realistic  ○ | Very realistic  ○ |
| Not at all useful  ○ | Not useful  ○ | Neutral  ○ | Useful  ○ | Very useful  ○ |
| Not at all interesting  ○ | Not interesting  ○ | Neutral  ○ | Interesting  ○ | Very interesting  ○ |
| Not at all true  ○ | Untrue  ○ | Neutral  ○ | True  ○ | Very true  ○ |
| Very exaggerated  ○ | Exaggerated  ○ | Neutral  ○ | Not exaggerated  ○ | Not at all exaggerated  ○ |

**To what extent do you agree with the information on the** website?

| I completely disagree  ○ | I disagree  ○ | Neutral  ○ | I agree  ○ | I completely agree  ○ |
| --- | --- | --- | --- | --- |

**1b. Can you provide a report mark for the website you just visited? (1 = very bad and 10 = very good)……….**

**Maastricht University is currently developing a website called ‘Zelftestwijzer’ (Self-test Guide). If you’d like to, you can indicate below what you thought was good or bad about the site you just visited. (Optional)**

**1c. What did you think was good about the website?…**

**1d. What did you think was bad about the website?......**

**Text intended only for control group**

**Some time ago you filled in a questionnaire in which you indicated that you would [Dynamic representation: perhaps, probably, definitely] want to do a cholesterol self-test in the future. That is what this questionnaire is about. Before you start filling in the questionnaire, we want to invite you to read a short text about self-tests**

**The link shown below will open the text in a new window. After having read the text, you can close the window and can start filling in the questionnaire by clicking the “Next” button below.**

**Please click the link shown below to go to the website.**

**Link**

**All respondents**

**The following questions are about self-tests (NOTE: this concerns all types of self-tests for cholesterol: self-tests for home use, tests done at a facility or tests done at a laboratory)**

**2a. Do you intend to use a cholesterol self-test in the future?**

- Definitely not (respondent is referred to question 3a)
- Probably not (respondent is referred to question 3a)
- Perhaps
- Probably
- Definitely

**2b. When would you [perhaps / probably / definitely, tailored on the basis of question 2a] want to do this self-test?**

- Within the next month
- Within the next 6 months (but not within the next month)
- Within the next year (but not within the next 6 months)
- Within the next 5 years (but not within the next year)
- Some time in the future (but not within the next 5 years)
- None of the above

**2c. What type(s) of self-test would you consider? (multiple answers allowed)**

- A self-test for home use
- Visiting a facility, having a test done there, and getting the results immediately
- Visiting a laboratory to have a body sample taken, and getting the results sent to me by post
- Sending in a body sample to a laboratory, and getting the results sent to me by post

**3a. Do you intend to ask your family doctor to do a cholesterol test in the future?**

- - Certainly not
  - Probably not
  - Possibly
  - Probably
  - Certainly

**3b. Do you intend to adapt your lifestyle in the future (e.g. eat more healthy, stop smoking, exercise more often)?**

- Certainly not (respondent is referred to question 4)
- Probably not (respondent is referred to question 4)
- Possibly
- Probably
- Certainly

**If 3b Possibly – Certainly:**

**3c. What do you intend to adapt according to your lifestyle? (multiple answers allowed)**

- Stop smoking
- Eat more healthy
- Exercise more often
- Take more rest / avoid stress
- Drink less alcohol
- Different, namely…..
- None of the above

**We now present a number of statements about self-tests for cholesterol. What we want to know is you personal opinion, what you personally think about it. Please click the option that corresponds most closely to your opinion.**

**4. To me, a cholesterol** self-test is….

| Harmful | ○ | ○ | ○ | ○ | ○ | ○ | ○ | Beneficial |
| --- | --- | --- | --- | --- | --- | --- | --- | --- |
| Unimportant | ○ | ○ | ○ | ○ | ○ | ○ | ○ | Important |
| Bad thing | ○ | ○ | ○ | ○ | ○ | ○ | ○ | Good thing |
| Unpleasant | ○ | ○ | ○ | ○ | ○ | ○ | ○ | Pleasant |

**5. The following questions concern the way you feel about a cholesterol self-test**

| **As regards doing a cholesterol self-test I have …….** | | | | | | | | |
| --- | --- | --- | --- | --- | --- | --- | --- | --- |
| Very definite feelings | ○ | ○ | ○ | ○ | ○ | ○ | ○ | Very mixed feelings |
| **As regards doing a cholesterol self-test I experience ….** | | | | | | | | |
| No conflicting feelings at all | ○ | ○ | ○ | ○ | ○ | ○ | ○ | Highly conflicting feelings |
| **As regards doing a diabetes self-test I feel…..** | | | | | | | | |
| No doubts at all | ○ | ○ | ○ | ○ | ○ | ○ | ○ | Very serious doubts |

**6. To what extent do you agree with the following statements concerning a cholesterol self-test?**

|  | Completely disagree | disagree | neutral | agree | Completely agree |
| --- | --- | --- | --- | --- | --- |
|  |  |  |  |  |  |
| If I do a cholesterol self-test, I think the result will be reliable | ○ | ○ | ○ | ○ | ○ |
| If my test result is normal (nothing’s the matter), I can be sure that this result is correct | ○ | ○ | ○ | ○ | ○ |
| If my test result is abnormal (something’s the matter), I can be sure that this result is correct | ○ | ○ | ○ | ○ | ○ |
| If the test result of my cholesterol self-test indicates that something’s the matter, I’m able to take the correct subsequent action | ○ | ○ | ○ | ○ | ○ |
|  |  |  |  |  |  |
| Performing a cholesterol self-test is difficult | ○ | ○ | ○ | ○ | ○ |
| When performing a cholesterol self-test, I would like to have professional assistance | ○ | ○ | ○ | ○ | ○ |

**We now present a number of statements about the self-test you would like to do in the future and the risk factor or disease it may concern.** **What we want to know is your personal opinion, what you personally think about it. Please click the option that corresponds most closely to your opinion..**

**7. To what extent do you agree with the following statements**?

|  | Completely disagree | Disagree | neutral | Agree | Completely agree |
| --- | --- | --- | --- | --- | --- |
|  |  |  |  |  |  |
| According to me, performing a cholesterol self-test is important | ○ | ○ | ○ | ○ | ○ |
| Self-testing for cholesterol means taking your own responsibility | ○ | ○ | ○ | ○ | ○ |
| Self-testing for cholesterol provides a sense of security about your own health | ○ | ○ | ○ | ○ | ○ |
| An important advantage of a cholesterol self-test is privacy | ○ | ○ | ○ | ○ | ○ |
| An important advantage of a cholesterol self-test is a fast result | ○ | ○ | ○ | ○ | ○ |
| An important advantage of a cholesterol self-test is that it saves time | ○ | ○ | ○ | ○ | ○ |
| By testing myself for cholesterol, I can reassure myself | ○ | ○ | ○ | ○ | ○ |
| By testing myself for cholesterol, I take care of my own health | ○ | ○ | ○ | ○ | ○ |
| It feels good to take responsibility for my own health | ○ | ○ | ○ | ○ | ○ |
|  |  |  |  |  |  |
| The costs of a cholesterol self-test are a barrier to me | ○ | ○ | ○ | ○ | ○ |
| Testing myself for cholesterol would make me too concerned with my health | ○ | ○ | ○ | ○ | ○ |
| Being (too) much concerned with my health scares me | ○ | ○ | ○ | ○ | ○ |
| Just thinking about cholesterol self-testing scares me | ○ | ○ | ○ | ○ | ○ |
| Just thinking about cholesterol self-testing makes me insecure | ○ | ○ | ○ | ○ | ○ |
|  | ○ | ○ | ○ | ○ | ○ |
| I would regret it if I didn’t perform this self-test and it subsequently appeared that I have an elevated cholesterol level | ○ | ○ | ○ | ○ | ○ |
|  |  |  |  |  |  |
| I perceive it as a moral obligation to myself to perform a cholesterol self-test | ○ | ○ | ○ | ○ | ○ |
| I perceive it as a moral obligation to the people around me to perform a cholesterol self-test | ○ | ○ | ○ | ○ | ○ |
|  |  |  |  |  |  |
| My partner (or others in my immediate environment) expects me to perform a cholesterol self-test | ○ | ○ | ○ | ○ | ○ |

**8. According to you, what are the chances that you will develop a cardiovascular disease? (cardiovascular diseases are for instance: heart attack, cerebral infarction, CVA, stroke, vascular constriction, claudication, percutaneous angioplasty)**

- Very high
- High
- Not high / not low
- Low
- Very low
- I already have cardiovascular disease (respondents are referred to question 11)

**9. according to you, what are the chances that you will develop cardiovascular disease compared to others of your age and gender?**

- Much larger
- Larger
- Equally large / small
- Smaller
- Much smaller

**10. To what extent do you feel worried in developing cardiovascular disease in the future?**

- Not worried at all
- Not worried
- Neutral
- Worried
- **Very worried**

**11. How severe do you think cardiovascular diseases are?**

- Very severe
- Severe
- Neutral
- Not severe
- Not severe at all

**12. Has anyone in your immediate environment had a cardiovascular disease?**

- No
- Yes

**13. To me, a reason to use a cholesterol self-test would** be:

|  | Completely disagree | Disagree | neutral | Agree | Completely agree |
| --- | --- | --- | --- | --- | --- |
|  |  |  |  |  |  |
| If I have a medical complaint | ○ | ○ | ○ | ○ | ○ |
| If I am worried that I might have a disease |  |  |  |  |  |
| If other people advise me to take the test | ○ | ○ | ○ | ○ | ○ |
| If the media advised me to take the test | ○ | ○ | ○ | ○ | ○ |
| If people in my immediate environment have the disease | ○ | ○ | ○ | ○ | ○ |
| If I wanted to know more about my health status | ○ | ○ | ○ | ○ | ○ |
| If the test was offered to me (free of charge) | ○ | ○ | ○ | ○ | ○ |

**14. Consider the statements below about cholesterol self-testing. Please indicate whether each statement is true or false. If you are unsure, you can also tick ‘Do not know’**.

|  | **True** | **False** | **Do not know** |
| --- | --- | --- | --- |
| The body needs cholesterol in order to function |  |  |  |
| *There are two types of cholesterol. One is the good cholesterol, the other the bad.*  Question: The HDL cholesterol is the bad cholesterol |  |  |  |
| In the Netherlands the most common cause of high cholesterol is unhealthy food |  |  |  |
| High cholesterol has physical symptoms that can be recognised |  |  |  |
|  | **True** | **False** | **Do not know** |
| People with high cholesterol are more likely to develop cardiovascular disease |  |  |  |
| If your cholesterol is normal (not higher than normal), then you have no increased risk of getting a cardiovascular disease. |  |  |  |
|  | **True** | **False** | **Do not know** |
| The next questions are about a home cholesterol test (a test that can be performed by yourself at home): |  |  |  |
| A home cholesterol test involves you taking a blood sample though a finger prick |  |  |  |
| A home cholesterol test that measures “total cholesterol”, will also indicate the good and bad cholesterol in the results |  |  |  |
| Cholesterol test kits can be stored and used indefinitely. |  |  |  |
|  | **True** | **False** | **Do not know** |
| If your blood cholesterol is higher than normal, it means that you have a cardiovascular disease |  |  |  |
| If high cholesterol is treated with a healthy diet, no medication is needed |  |  |  |
| If total cholesterol is normal (not elevated), the level of bad cholesterol is also normal |  |  |  |
| *Ms. Jansen, 67 years old, decides to carry out a self-test for cholesterol. The result is normal. What does this mean?*  Answer: This means that Ms. Jansen can be reassured, but only if she has no other risk factors for cardiovascular disease. |  |  |  |
| *Mr. Gerritsen, 58 years old, decides to carry out a self-test for cholesterol because he often eats unhealthy food. The result is abnormal (elevated cholesterol). What should Mr. Gerritsen do now?*  Answer: Mr. Gerritsen should go see his doctor to have his cardiovascular risk assessed |  |  |  |
|  | **True** | **False** | **Do not know** |
| Sometimes a test indicates that cholesterol is elevated, while in fact it is normal. |  |  |  |
| A false, or unjustified normal or abnormal result can occur even if the test is carried out according to instructions |  |  |  |
| *The following issues affect the accuracy of the test result of a home cholesterol test:* |  |  |  |
| How the test is stored |  |  |  |
| The amount of blood that you use in the test |  |  |  |
| Which finger blood is taken from |  |  |  |
| Looking at the results in sunlight |  |  |  |

**15. While answering the above questions, did you look for information elsewhere? (If yes, multiple answers are allowed)**

- no

- yes, on the Internet

- yes, in a book, magazine or brochure

- yes, asked a relative or friend

- yes, other source(s)

**16. Would you allow the Maastricht University researchers to perhaps contact you later for an interview, to be held at the University or at your home, for which you would receive a financial remuneration? If so, could you please enter a telephone number or email address where the researchers could reach you.**

○ no

○ yes, my phone number is …

○ yes, my email address is …

# This is the end of the questionnaire.

**Thank you very much for your cooperation!**

**Please click NEXT to submit the questionnaire.**
