# Supplementary material for: A single-blind randomised controlled trial of the effects of a web-based decision aid on self-testing for cholesterol and diabetes. study protocol
Source: BMC Public Health. 2012 Jan 4;12:6. doi: 10.1186/1471-2458-12-6 (PMC3298527; doi:10.1186/1471-2458-12-6)
Supplement: Additional file 3 — Questionnaire 2 Diabetes (translated from Dutch). [file 1471-2458-12-6-S3.PDF]

Second questionnaire Diabetes

The questionnaire was originally in Dutch and has been translated into English

Text and question 1 only for intervention group

Some time ago you filled in a questionnaire in which you indicated that you would [Dynamic representation: perhaps, probably, definitely] want to do a diabetes self-test in the future. That is what this questionnaire is about. Before you start filling in the questionnaire, we want to invite you to visit a website.

The link shown below will open the site in a new window. After having visited the site, you can close the window and can start filling in the questionnaire by clicking the "Next" button below.

Please click the link shown below to go to the website.

Link

You have just been shown some information about self-tests. This questionnaire presents you with some questions about the information you have just seen. It also asks you some questions about diabetes self-tests and certain aspects that may relate to it.

1a. Do you think the information on the website was….

Not at all convincing ○	Not convincing ○	Neutral ○	convincing ○	Very convincing ○
Not at all relevant ○	Not relevant ○	Neutral ○	Relevant ○	Very relevant ○
Not at all realistic ○	Not realistic ○	Neutral ○	Realistic ○	Very realistic ○
Not at all useful ○	Not useful ○	Neutral ○	Useful ○	Very useful ○
Not at all interesting ○	Not interesting ○	Neutral ○	Interesting ○	Very interesting ○
Not at all true ○	Untrue ○	Neutral ○	True ○	Very true ○
Very exaggerated ○	Exaggerated ○	Neutral ○	Not exaggerated ○	Not at all exaggerated ○

To what extent do you agree with the information on the website?

I completely disagree ○	I disagree ○	Neutral ○	I agree ○	I completely agree ○

1b. Can you provide a report mark for the website you just visited? (1 = very bad and 10 = very good)……….

Maastricht University is currently developing a website called 'Zelftestwijzer' (Self-test Guide). If you'd like to, you can indicate below what you thought was <u>good</u> or <u>bad</u> about the site you just visited. (Optional)

1c. What did you think was good about the website?…

1d. What did you think was bad about the website?......

Text intended only for control group

Some time ago you filled in a questionnaire in which you indicated that you would [Dynamic representation: perhaps, probably, definitely] want to do a diabetes self-test in the future. That is what this questionnaire is about. Before you start filling in the questionnaire, we want to invite you to read a short text about self-tests

The link shown below will open the text in a new window. After having read the text, you can close the window and can start filling in the questionnaire by clicking the “Next” button below.

Please click the link shown below to go to the website.

Link

All respondents
The following questions are about self-tests (NOTE: this concerns all types of self-tests for diabetes: self-tests for home use, tests done at a facility or tests done at a laboratory)

2a. Do you intend to use a diabetes self-test in the future?
- ☐ Definitely not (respondent is referred to question 3a)
- ☐ Probably not (respondent is referred to question 3a)
- ☐ Perhaps
- ☐ Probably
- ☐ Definitely

2b. When would you [perhaps / probably / definitely, tailored on the basis of question 2a] want to do this self-test?
- ☐ Within the next month
- ☐ Within the next 6 months (but not within the next month)
- ☐ Within the next year (but not within the next 6 months)
- ☐ Within the next 5 years (but not within the next year)
- ☐ Some time in the future (but not within the next 5 years)
- ☐ None of the above

2c. What type(s) of self-test would you consider? (multiple answers allowed)
- ☐ A self-test for home use
- ☐ Visiting a facility, having a test done there, and getting the results immediately
- ☐ Visiting a laboratory to have a body sample taken, and getting the results sent to me by post
- ☐ Sending in a body sample to a laboratory, and getting the results sent to me by post

3a. Do you intend to ask your family doctor to do a diabetes test in the future?

- Certainly not
- Probably not
- Possibly
- Probably
- Certainly

3b. Do you intend to adapt your lifestyle in the future (e.g. eat more healthy, stop smoking, exercise more often)?

- Certainly not (respondent is referred to question 4)
- Probably not (respondent is referred to question 4)
- Possibly
- Probably
- Certainly

If 3b Possibly – Certainly:

3c. What do you intend to adapt according to your lifestyle? (multiple answers allowed)

- Stop smoking
- Eat more healthy
- Exercise more often
- Take more rest / avoid stress
- Drink less alcohol
- Different, namely…..
- None of the above

We now present a number of statements about self-tests for diabetes. What we want to know is you personal opinion, what you personally think about it. Please click the option that corresponds most closely to your opinion.

4. To me, a diabetes self-test is….

Harmful	○	○	○	○	○	○	○	Beneficial
Unimportant	○	○	○	○	○	○	○	Important
Bad thing	○	○	○	○	○	○	○	Good thing
Unpleasant	○	○	○	○	○	○	○	Pleasant

5. The following questions concern the way you feel about a diabetes self-test

**As regards doing a diabetes self-test I have …….**								
Very definite feelings	○	○	○	○	○	○	○	Very mixed feelings
**As regards doing a diabetes self-test I experience ….**								
No conflicting feelings at all	○	○	○	○	○	○	○	Highly conflicting feelings
**As regards doing a diabetes self-test I feel…..**								
No doubts at all	○	○	○	○	○	○	○	Very serious doubts

6. To what extent do you agree with the following statements concerning a diabetes self-test?

	Completely disagree	disagree	neutral	agree	Completely agree
If I do a diabetes self-test, I think the result will be reliable	○	○	○	○	○
If my test result is normal (nothing's the matter), I can be sure that this result is correct	○	○	○	○	○
If my test result is abnormal (something's the matter), I can be sure that this result is correct	○	○	○	○	○
If the test result of my diabetes self-test indicates that something's the matter, I'm able to take the correct subsequent action	○	○	○	○	○
Performing a diabetes self-test is difficult	○	○	○	○	○
When performing a diabetes self-test, I would like to have professional assistance	○	○	○	○	○

We now present a number of statements about <u>the self-test you would like to do in the future and the risk factor or disease it may concern</u>. What we want to know is your personal opinion, what you personally think about it. Please click the option that corresponds most closely to your opinion.

7. To what extent do you agree with the following statements?

	Completely disagree	Disagree	neutral	Agree	Completely agree
According to me, performing a diabetes self-test is important	○	○	○	○	○
Self-testing for diabetes means taking your own responsibility	○	○	○	○	○
Self-testing for diabetes provides a sense of security about your own health	○	○	○	○	○
An important advantage of a diabetes self-test is privacy	○	○	○	○	○
An important advantage of a diabetes self-test is a fast result	○	○	○	○	○
An important advantage of a diabetes self-test is that it saves time	○	○	○	○	○
By testing myself for diabetes, I can reassure myself	○	○	○	○	○
By testing myself for diabetes, I	○	○	○	○	○

take care of my own health					
It feels good to take responsibility for my own health	○	○	○	○	○
The costs of a diabetes self-test are a barrier to me	○	○	○	○	○
Testing myself for diabetes would make me too concerned with my health	○	○	○	○	○
Being (too) much concerned with my health scares me	○	○	○	○	○
Just thinking about diabetes self-testing scares me	○	○	○	○	○
Just thinking about diabetes self-testing makes me insecure	○	○	○	○	○
	○	○	○	○	○
I would regret it if I didn't perform this self-test and it subsequently appeared that I have diabetes	○	○	○	○	○
I perceive it as a moral obligation to myself to perform a diabetes self-test	○	○	○	○	○
I perceive it as a moral obligation to the people around me to perform a diabetes self-test	○	○	○	○	○
My partner (or others in my immediate environment) expects me to perform a diabetes self-test	○	○	○	○	○

8. According to you, what are the chances that you will develop diabetes?

- ☐ Very high
- ☐ High
- ☐ Not high / not low
- ☐ Low
- ☐ Very low
- ☐ I already have cardiovascular disease (respondents are referred to question 11)

9. according to you, what are the chances that you will develop diabetes compared to others of your age and gender?

- ☐ Much larger
- ☐ Larger
- ☐ Equally large / small
- ☐ Smaller
- ☐ Much smaller

10. To what extent do you feel worried in developing diabetes in the future?

- Not worried at all
- Not worried
- Neutral
- Worried
- Very worried

11. How severe do you think diabetes is?

- Very severe
- Severe
- Neutral
- Not severe
- Not severe at all

12. Does anyone in your immediate environment have diabetes?

- No
- Yes

13. To me, a reason to use a diabetes self-test would be:

	Completely disagree	Disagree	neutral	Agree	Completely agree
If I have a medical complaint	○	○	○	○	○
If I am worried that I might have a disease					
If other people advise me to take the test	○	○	○	○	○
If the media advised me to take the test	○	○	○	○	○
If people in my immediate environment have the disease	○	○	○	○	○
If I wanted to know more about my health status	○	○	○	○	○
If the test was offered to me (free of charge)	○	○	○	○	○

14. Consider the statements below about diabetes self-testing. Please indicate whether each statement is true or false. If you are unsure, you can also tick 'Do not know'.

	**True**	**False**	**Do not know**
In diabetes, the glucose level in the blood is too high			
You can have diabetes without being aware of it.			
A common symptom of diabetes is feeling more thirsty than usual			
People who have diabetes are more likely to have nerve damage			
	**True**	**False**	**Do not know**
There are some factors that may increase or decrease the risk of developing diabetes			
If you are overweight, you are more likely to get diabetes			
If you have a family history of diabetes, you are more likely to get diabetes			

Smoking lowers your chance of developing diabetes			
	**True**	**False**	**Do not know**
There are different types of home testing for diabetes (tests you can perform yourself at home). There are tests that use blood, or tests for which urine must be collected.			
A home diabetes test involves you taking a blood sample though a finger prick			
The home diabetes test is as good at detecting diabetes as the home blood test			
The time at which you perform the test (e.g. in the morning or at bedtime) is important.			
Diabetes test kits can be stored and used indefinitely.			
	**True**	**False**	**Do not know**
Mr Jones, aged 67 years, decides to carry out a self-test for diabetes. Mr Jones has followed the test instructions and completed the test twice. Both test results were normal.<br><br>Answer: This means that Mr Jones does NOT have diabetes			
Ms. De Boer, aged 64 years, decides to carry out a self-test for diabetes because she has problems when urinating. The result of the test was normal.<br><br>Answer: Ms de Boer should now wait and repeat the test in 1 month.			
Mr. Gerritsen, 58 years old, decides to carry out a self-test for diabetes. The test result was abnormal. In accordance with the self-test recommendations, Ms Gerritsen should now see her doctor to discuss the results.			
	**True**	**False**	**Do not know**
Sometimes a test indicates that the glucose level is elevated, while in fact it is normal.			
A false, or unjustified normal or abnormal result can occur even if the test is carried out according to instructions			
The following issues affect the accuracy of the test result of a home diabetes test:			
How the test is stored			
The amount of blood that you use in the test			
Which finger blood is taken from			
Looking at the results in sunlight			

15. While answering the above questions, did you look for information elsewhere? (If yes, multiple answers are allowed)
- no
- yes, on the Internet
- yes, in a book, magazine or brochure
- yes, asked a relative or friend
- yes, other source(s)

16. Would you allow the Maastricht University researchers to perhaps contact you later for an interview, to be held at the University or at your home, for which you would receive a financial remuneration? If so, could you please enter a telephone number or email address where the researchers could reach you.

○ no
○ yes, my phone number is …
○ yes, my email address is …

This is the end of the questionnaire.
Thank you very much for your cooperation!

Please click NEXT to submit the questionnaire.
