# Supplementary material for: A single-blind randomised controlled trial of the effects of a web-based decision aid on self-testing for cholesterol and diabetes. study protocol
Source: BMC Public Health. 2012 Jan 4;12:6. doi: 10.1186/1471-2458-12-6 (PMC3298527; doi:10.1186/1471-2458-12-6)
Supplement: Additional file 4 — Questionnaire 3 (translated from Dutch). [file 1471-2458-12-6-S4.PDF]

Third questionnaire

The questionnaire was originally in Dutch and has been translated into English

Some time ago you filled in a questionnaire in which you indicated that you might want to do a … [dynamic representation] self-test in the future. We would like to ask you some further questions.

1. In the past 3 months, did you do a self-test (that is, a test on your blood or urine which you did or had done, without a doctor being involved)? (if yes, multiple answers allowed)

- Yes, a diabetes self-test
- Yes, a cholesterol self-test
- Yes, another self-test
- No

2. Did the information you received about self-tests cause you to consult a doctor during the past 3 months?

- Yes
- No

3. Did the information you received about self-tests cause you to seek other professional help (such as a nurse or nurse practitioner)?

- Yes
- No

4. Did the information you received about self-tests cause you to change your lifestyle in the past 3 months? (multiple answers allowed)

- Yes, I've started to take more exercise
- Yes, I've stopped smoking
- Yes, I've started to eat healthier food
- Yes, I've reduced my alcohol intake
- Yes, other: ……
- No

If yes is ticked at question 1, respondent is referred to question 5 for each test (diabetes or cholesterol); if no or 'another test' is ticked at question 1, respondent is referred to question 6.

5a. What was de main reason for you to do a [dynamic representation] self-test?

- Because I had a medical complaint
- Because I was worried I might have a disease
- Because other people advised me to take the test
- Because the test was recommended in a campaign
- Because people in my immediate environment had the disease
- Because I had had the test before and wanted to know if anything had changed
- Because I wanted to know more about my health status
- Because the test was offered to me (free of charge)
- Other, namely ………………………………..

5b. Why did you decide to take a self-test instead of visiting your family doctor? (multiple answers allowed)

- Because such a test ensures privacy
- Because you get the results quickly
- Because the test was offered to me (free of charge)
- Because I wanted to take responsibility for my own health
- Because I previously asked my doctor to do the test but it was never done
- Other, namely…………………………………………………..

5c. What was the result of the self-test?

- Normal (nothing wrong)
- Abnormal (something wrong)
- Inconclusive
- Test failed
- Can't remember
- I prefer not to answer this question

5d. Were you reassured by the result of this self-test?

- Not at all reassured
- Not reassured
- Neutral
- Reassured
- Completely reassured

5e. What did you do after you got the test result? *(multiple answers allowed)*

- I took no further action.
- I discussed the test result with relatives or friends.
- I looked for further information.
- I did a self-test for other diseases or risk factors.
- I changed my lifestyle.
- I bought over-the-counter drugs (such as vitamins or pills).
- I sought help from complementary medicine (such as acupuncture or homeopathy).
- I consulted a doctor.
- I consulted another care provider (such as a dietician, a psychologist, a physiotherapist, my GP's receptionist or a nurse practitioner).
- Other

5f. You indicated that you might want to do a [dynamic representation] self-test in the future, and you have since actually done so. Would you do another [dynamic representation] self-test in the future?

- Definitely not (respondent is referred to question 7)
- Probably not (respondent is referred to question 7)
- Perhaps
- Probably
- Definitely

If the Perhaps is ticked at question 5f, respondent is referred to question 5g.

5g. When would you [perhaps / probably / definitely; tailored on the basis of question 5a) think of doing this self-test?

- Within the next month
- Within the next 6 months (but not within the next month)
- Within the next year (but not within the next 6 months)
- Within the next 5 years (but not within the next year)
- Some time in the future (but not within the next 5 years)
- None of the above

5h. According to you, what are the chances that you will develop …. [dynamic representation]?

- Very high
- High
- Not high / not low
- Low
- Very low
- I already have diabetes (respondents are referred to question 11)

5i. According to you, what are the chances that you will develop …. [dynamic representation] compared to others of your age and gender?

- Much larger
- Larger
- Equally large / small
- Smaller
- Much smaller

5j. To what extent do you feel worried about developing [dynamische weergave] in the future?

- Not worried at all
- Not worried
- Neutral
- Worried
- Very Worried

Only for those respondents who ticked No or 'other test' at question 1

6a. You indicated before that you might want to do a [dynamic representation] self-test in the future. Do you still intend to do this self-test in the future?

- Definitely not (respondent is referred to question 7)
- Probably not (respondent is referred to question 7)
- Perhaps
- Probably
- Definitely

If Perhaps–Definitely is ticked at question 6a, respondent is referred to question 6b.

6b. When would you want to do this self-test?

- Within the next month

- Within the next 6 months (but not within the next month)
- Within the next year (but not within the next 6 months)
- Within the next 5 years (but not within the next year)
- Some time in the future (but not within the next 5 years)
- None of the above

All respondents:

7. Do you intend to consult a doctor because you have questions about [dynamic representation]?

- ☐ Definitely not
- ☐ Probably not
- ☐ Perhaps
- ☐ Probably
- ☐ Definitely

This is the end of the questionnaire.
Thank you very much for your cooperation!

Please click NEXT to submit the questionnaire.
